# Supplementary material for: Relationship between Bacterial Vaginosis and Sexually Transmitted Infections: Coincidence, Consequence or Co-Transmission?
Source: Microorganisms. 2023 Oct 1;11(10):2470. doi: 10.3390/microorganisms11102470 (PMC10609101; doi:10.3390/microorganisms11102470)
Supplement: Supplementary file 1 [file microorganisms-11-02470-s001.zip › microorganisms-2552044-supplementary.pdf]

**Supplementary Table S1.** Table of primers and probes used to detect microorganisms involved in bacterial vaginosis and sexually transmitted infections.

| Targets | Targeted sequences | Primer names | Sequences (5'-3') | Probes |
|---|---|---|---|---|
| *Gardnerella vaginalis* | *Cpn 60* | Gvaginalis_F | CGCATCTGCTAAGGATGTTG | VIC-TGCAACTATTTCTGCAGCAGATCCT |
| | | Gvaginalis_R | CAGCAATCTTTTCGCCAACT | |
| *Fannyhessea* (*Atopobium*) *vaginae* | 16S rRNA | Atop_F | CCCTATCCGCTCCTGATACC | VIC-GCAGGCTTGAGTCTGGTAGGGGA |
| | | Atop_R | CCAAATATCTGCGCATTTCA | |
| *Lactobacillus* spp | *Tuf* | Lacto_F2 | TACATYCCAACHCCAGAACG | FAM-AAGCCATTCTTRATGCCAGTTGAA |
| | | Lacto_R2 | AAGCAACAGTACCACGACCA | |
| Human albumin | Exon 12 | Alb_F | GCTGTCATCTCTTGTGGGCTGT | FAM-CCTGTCATGCCCACACAAATCTCTCC |
| | | Alb_R | AAACTCATGGGAGCTGCTGGTTC | |
| *Mycoplasma genitalium* | *FusA* | Mgen_fusA_F | CTGGGAAAACCACCACATCA | FAM-TGGTGAATCAGTGATGGACTGGATGGA |
| | | Mgen_fusA_R | CACTGAAGTGGCTGCAGAGG | |
| *Trichomonas vaginalis* | Repeated sequence | Tvag_L23861_F | AAGATGGGTGTTTTAAGCTAGATAAGGT | FAM-CCGAAGTTCATGTCCTCTCCAAGCGT |
| | | Tvag_L23861_R | CGTCTTCAAGTATGCCCCAGTAC | |
| *Neisseria gonorrhoeae* | Hypothetical protein | Ngono_2F | CATCAGCGCAATCCCTATGA | FAM-CCACGGCGCTTCCTGATGGG |
| | | Ngono_2R | TGGTTGTCCTCAACCGTGTC | |
| *Chlamydia trachomatis* | Hypothetical protein | Ctracho_1F | AGCTCCCAAAGCAACCAGAR | FAM-CAACAGCACCACCAGCAGCTGC |
| | | Ctracho_1R | BTGTCGCTGCGTTGGTTTTA | |
| *Treponema pallidum* | *PolA* | SyphT_F1 | GTCGAGACTGAAAAGGAGTGCA | FAM-TGCTGTGCAGGATCCGGCATATGTCC |
| | | SyphT_R1 | GTGAGCGTCTCATCATTCCAAAG | |
| *Haemophilus ducreyi* | *GroESL* | H.duc_groE_F | CACAATGAGTATTCGTCCATTACAC | FAM-AGTGCTGCAATTCCAGATACCGATCGC |
| | | H.duc_groE_R | GCAATCACTTTACCGCGAGT | |
| Herpes simplex virus | *Pol* | HSV DNA POL F | CATCACCGACCCGGAGAGGGAC | FAM-CCGCCGAACTGAGCAGACACCCGCGC |
| | | HSV DNA POL R | GGGCCAGGCGCTTGTTGGTGTA | |

**Supplementary Table S2**. Review of the international literature presenting various arguments in favour or against sexual transmission of bacterial vaginosis.

| | **For the sexual transmission of bacterial vaginosis** | **Against the sexual transmission of bacterial vaginosis** |
|---|---|---|
| **Epidemiology** | | |
| **Bacterial transfer** | - Development of typical clinical manifestations of BV, presence of *G. vaginalis* in pure culture, and characteristical microscopic findings of stained smears in women, previously free of BV and *G. vaginalis*, directly inoculated with material from the vaginas of patients with BV suggesting the possibility of sexual inoculation (**First exhaustive study on "*Haemophilus vaginalis* vaginitis" in 1955** [41]).<br><br>- Enriched penile microbiome with taxa associated with BV of sex partners of women with BV → Sexual exchange of penile and vaginal taxa during intercourse (**1 study in 2020** [42]). | - Absence of microorganisms associated with BV in male partners of women with BV (**2 studies in 1984 and 1990** [43,44]). |
| **Antigen prostatic** | - Association of prostate-specific antigen, indicating recent semen exposure, with inflammatory responses in the female genital tract and the prevalence of BV (**1 study in 2021** [45]). | |
| **Male circumcision** | - Association of circumcision with a significant change in the overall microbiota and a significant decrease in putative anaerobic bacterial genera previously associated with BV (**4 studies in 2009-2020** [46–49]). | - No association found between the presence of BV in women and the circumcision status of their male partner (**1 study in 1999** [50]). |
| **Sexual behaviors** (Multiple partners, new partners, sexual practices) | - Protective effect of the consistent condom use against BV → Same protection method as for other STIs (**5 studies in 1955-2013** [41, 51–54])<br><br>- Prevalence of BV significantly associated with exposure to a new sexual partner, frequency of vaginal intercourse, unprotected intercourse, rectal intercourse, and use of lubricant → Similar sexual behavior between women with BV and those at risk for current STIs (**8 studies in 1955-2022** [7, 40,41, 55–60]).<br><br>- In relationships among women who have sex with women (WSW), certain sexual behaviors, including engaging with a new female partner, having a partner with BV symptoms, practicing oral-anal sex, and inadequate hygiene practices for insertable sex toys, can lead to concordant vaginal secretions in WSW and can impact on the vaginal microbiota causing changes in the community state types of the vaginal | - No significant difference in the prevalence of BV observed between the sexually active group and the virgin group (**2 studies in 1988 and 2010** [66, 67]).<br><br>- No association observed between any WSW sexual practices that would have the propensity to pass vaginal secretions from one to the other and BV (**1 study in 1999** [68]). |

| | | | | | | |
|---|---|---|---|---|---|---|
| | microbiota **(5 studies in 1995-2019** [61–65]). | | | | | |
| **Therapeutic** | **Treatment effectiveness: 6 studies in 1989-2021** | | | **Treatment ineffectiveness: 4 studies in 1988-1997** | | |
| Male treatment | **Size [References]** | **Treatment of women** | **Treatment arm for men** | **Size [References]** | **Treatment of women** | **Treatment arm for men** |
| | 342 couples [69] | Oral MTZ 500 mg bid 7d | Oral MTZ 500 mg bid and topical clindamycin bid 7d | 106 couples [70] | Oral MTZ 2 g on d1 and 3 | Oral MTZ 2 g on d1 and 3 |
| | 214 couples [71] | Oral MTZ 500 mg bid 7d | Oral MTZ 500 mg bid 7d | 190 couples [72] | Oral MTZ 2 g on d1 and 3 | Oral MTZ 2 g on d1 and 3 |
| | 16 couples [73] | Oral MTZ 400 mg bid 7d | Oral MTZ 400 mg bid and topical clindamycin bid 7d | 133 couples [74] | Oral TIN 2 g single dose | Oral TIN 2 g single dose |
| | 34 couples [75] | Oral MTZ 400 mg bid 7d | Oral MTZ 400 mg bid and topical clindamycin bid 7d | 139 couples [76] | 2% clindamycin intravaginal cream 7 nights | Oral clindamycin 150 mg qid |
| | 29 couples [77] | Oral MTZ 400 mg bid 7d | Oral MTZ 400 mg bid and topical clindamycin bid 7d | | | |
| | 161 couples [78] | Oral MTZ 2 g single dose OR Oral MTZ 500 mg bid 7d | Oral MTZ 2 g single dose OR Oral MTZ 500 mg bid 7d | | | |

* MTZ, metronidazole; TIN, tinidazole; d, days; Sample size reflects those included in the analysis.
